# Ischemic Stroke in a Talapoin Monkey ( *Miopithecus talapoin* )

**DOI:** 10.1111/jmp.70064

**Published:** 2026-02-02

**Authors:** Lucas dos Reis de Souza, Virginia Beatriz D’assunção Castro, Daniel Oliveira Santos, Nayara Ferreira de Paula, Paula Cristina Senra Lima, Rafael Otavio Cançado Motta, Carlyle Mendes Coelho, Herlandes Penha Tinoco, Rodrigo Otavio Silveira Silva, Renato Lima Santos, Ayisa Rodrigues de Oliveira

**Affiliations:** ^1^ Escola de Veterinária Universidade Federal de Minas Gerais Belo Horizonte Minas Gerais Brazil; ^2^ Fundação de Parques Municipais e Zoobotânica de Belo Horizonte Belo Horizonte Minas Gerais Brazil

**Keywords:** arteriosclerosis, infarct, malacia, thrombosis

## Abstract

A captive senile talapoin monkey ( *Miopithecus talapoin* ) developed sudden neurological signs and died within 24 h. Necropsy revealed an extensive infarct in the right hemisphere, from occipital to frontal lobe. Microscopy showed a thrombus, neuronal edema, and necrosis, and arteriosclerosis, characterizing an ischemic stroke.

## 1 Introduction

Stroke is associated with neuronal necrosis and irreversible loss of neurological function caused by ischemia. It may be classified as either ischemic or hemorrhagic depending on the underlying cause [1]. Ischemic strokes may result from vasculopathies such as arteriosclerosis, atherosclerosis, or vasculitis, as well as from thromboembolic disorders such as valvular endocarditis and atrial fibrillation and are grossly characterized by extensive areas of infarction [1]. Conversely, hemorrhagic strokes result from vascular rupture and can be caused by hypertension, arteriovenous fistulas, venous distension, aneurysms, or vascular wall amyloidosis, among other conditions. It is characterized by the extravasation of blood into the meninges or parenchyma, resulting in extensive hemorrhage [1]. Nonhuman primates, particularly Old‐World species, are often employed as experimental models for human stroke studies; however, reports of naturally occurring disease in these animals are scarce [2, 3, 4, 5]. This report aims to describe a case of naturally occurring ischemic stroke in a senile talapoin monkey ( *Miopithecus talapoin* ).

## 2 Case Report

All procedures strictly adhered to humane care of animals and all applicable laws and regulations as well as the ethical policies of the journal.

A 30‐year‐old male talapoin monkey ( *Miopithecus talapoin* ), housed at the Belo Horizonte Zoo (State of Minas Gerais, Brazil), was found exhibiting acute neurological signs, including anisocoria, horizontal nystagmus, flaccidity of the left limbs, loss of pain perception and motor strength, and mandibular contraction. The animal was taken to the zoo veterinary hospital for supportive treatment but died the following day, approximately 24 h after the onset of clinical signs. The carcass was sent for necropsy at the Universidade Federal de Minas Gerais.

**FIGURE 1:**
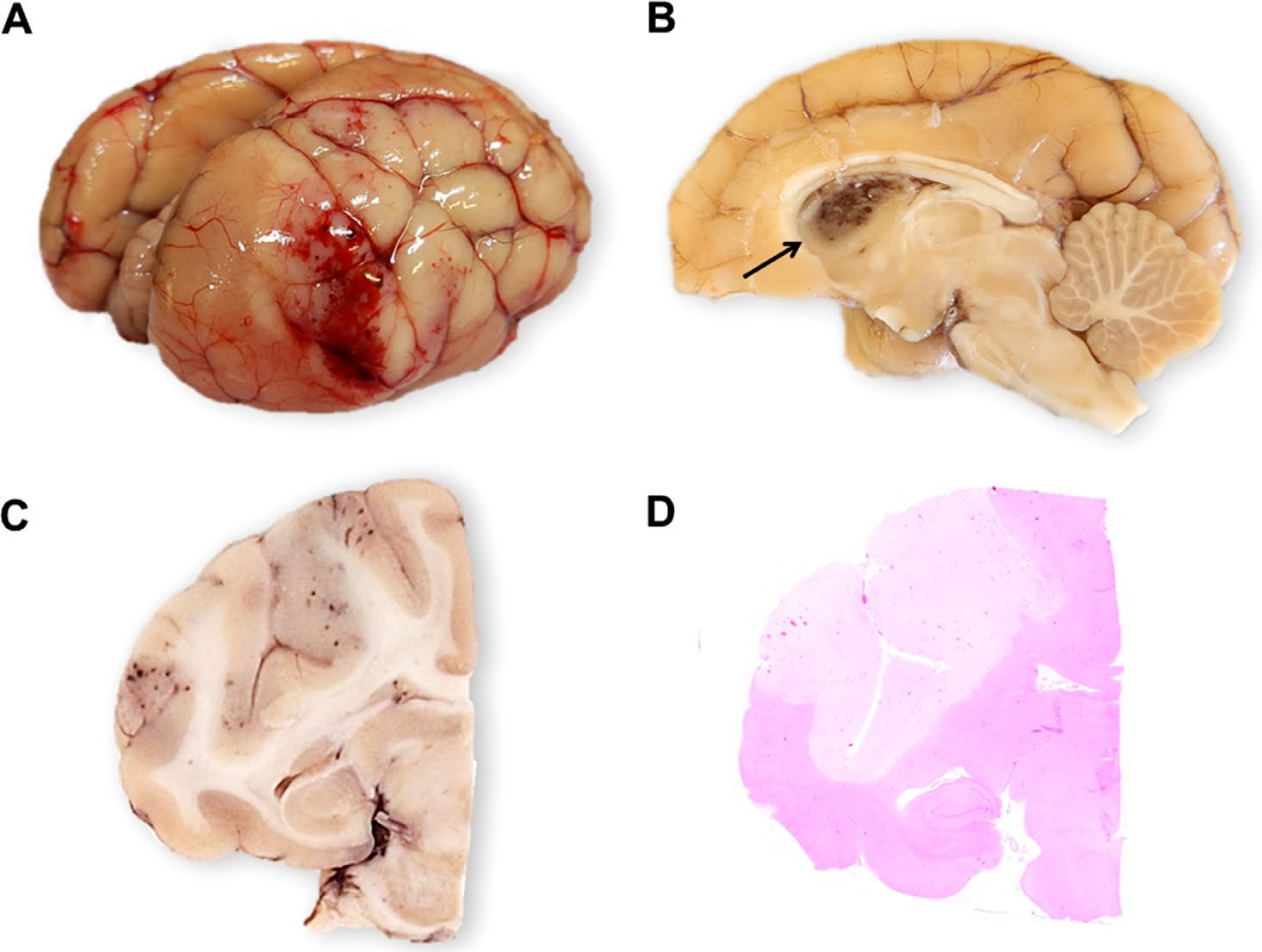
Ischemic stroke in talapoin monkey ( *Miopithecus talapoin* ). (A) Brain, right hemisphere with focally extensive pale area extending from the occipital to the frontal lobe, with irregular borders and areas of petechiae and suffusions in the leptomeninges, predominantly in the occipital region. (B) Brain, right hemisphere fixed in formalin, arrow shows areas of hemorrhage and malacia in the thalamus. (C) Brain, telencephalic cortex extending into white matter with focally extensive area of malacia with congestion of adjacent blood vessels. (D) Brain, subgross view, the area corresponding to the gross lesion showed a focally extensive loss of hematoxylin and eosin staining affinity, with visible blood vessels, consistent with a focally extensive infarct.

Grossly, there was a focally extensive and well delimited pale area in the right hemisphere of the brain extending from the occipital to the frontal lobe, with irregular borders and areas of petechiae and suffusions in the leptomeninges, predominantly in the occipital region (Figure 1A). On the cut surface, there were areas of malacia and hemorrhage in the right thalamus (Figure 1B), and areas of malacia that extended through the telencephalic cortex to the white matter, with congested blood vessels adjacent to those areas (Figure 1C). The pale areas (interpreted as malacia) were soft compared to the unaffected brain tissue. After fixation in 10% buffered formalin, the entire brain was cut in sequential 0.5 cm‐thick sections (30 slices). Other relevant gross findings included moderate opacity of the left lens, mild hepatomegaly with moderate evidence of the centrilobular pattern, generalized congestion, and mild mitral valve endocardiosis.

**FIGURE 2:**
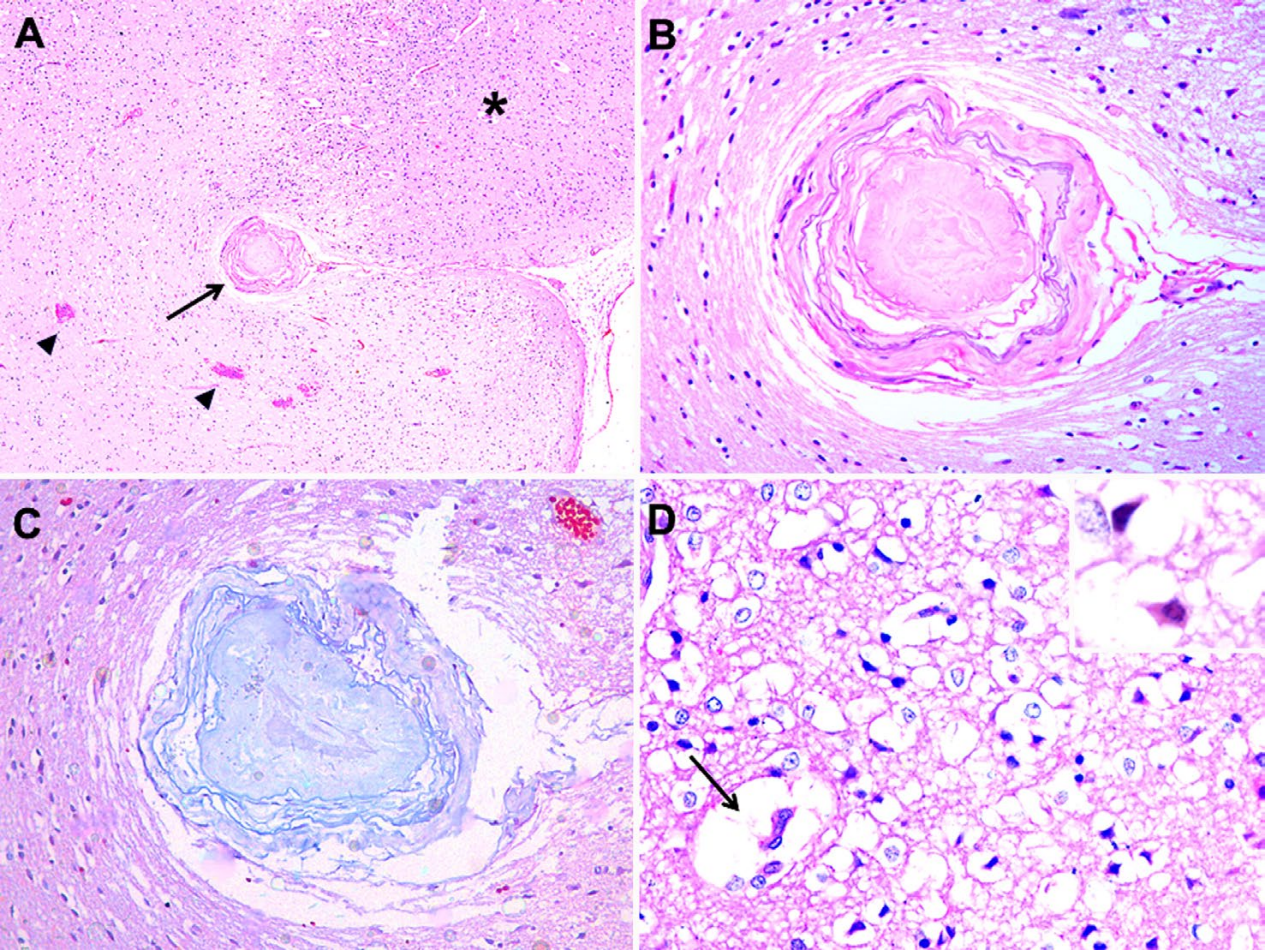
Ischemic stroke in talapoin monkey ( *Miopithecus talapoin* ) brain, right occipital lobe. (A) Arrow indicates a vessel in the Virchow‐Robin space completely occluded by amorphous eosinophilic material adherent to the endothelium (thrombus), with reduced staining affinity in adjacent neuroparenchyma at the necrotic‐viable tissue interface. The asterisk indicates normal tissue, and the arrowhead shows multifocal hemorrhages. HE, 40×. (B) Blood vessel with irregular wall and obstructed by thrombus. HE, 200×. (C) Blood vessel with irregular wall and obstructed by thrombus, the material obliterating the vessel is stained blue by Masson's trichrome staining, 200×. (D) Gray matter, the arrow indicates expansion of perivascular spaces (edema). There is diffuse neuropil vacuolization, retraction of glial cells and neurons, and triangular red neurons indicative of neuronal necrosis (inset). HE, 400×.

Histological examination of the brain revealed a focally extensive area of reduced staining affinity, which corresponded to the pale areas observed grossly (Figure 1D). In the occipital area, at the transition between necrotic and viable tissue, a vessel within the Virchow‐Robin space was completely occluded by amorphous, eosinophilic material adherent to the endothelium, with reduced staining affinity in the adjacent neuroparenchyma and multifocal hemorrhage (Figure 2A,B). The vessel wall was irregular and stained blue by Masson's trichrome (Figure 2C). Within the affected parenchyma, there was diffuse vacuolization of the neuropil, expansion of perivascular spaces (edema), and retraction of glial cells and neurons, with formation of triangular red neurons (indicative of neuronal necrosis) (Figure 2D). Multifocal areas of hemorrhage and a mild multifocal neutrophilic infiltrate were also observed. Gram staining did not reveal any intralesional bacteria. In other cerebral blood vessels, there was irregular deposition of amorphous eosinophilic material within the tunica media, with deposition of irregular granular eosinophilic material and occasional basophilic and birefringent granular deposits suggestive of mineralization. There was mild multifocal perivascular hemorrhage in the cervical spinal cord. Vascular changes suggestive of arteriosclerosis were also observed in other organs, including the lungs, heart, and spleen. Additional significant findings included moderate diffuse centrilobular hepatic necrosis with congestion, myxomatous degeneration of the mitral valve, mild multifocal glomerulosclerosis, and a cataract in the left lens. Samples of the areas of malacia and hemorrhage were sent for aerobic bacterial isolation, but no bacterial growth was observed.

## 3 Discussion

This report describes morphologic changes in the brain attributed to an ischemic stroke in a talapoin monkey ( *Miopithecus talapoin* ). The clinical and pathological findings of experimentally induced strokes in nonhuman primates are comparable to those reported in this case [2, 3, 4, 5]. Descriptions of spontaneous strokes in nonhuman primates are scarce. One study described the natural occurrence of the disease in six elderly female chimpanzees ( *Pan troglodytes* ) at a primatology center, reporting both ischemic and hemorrhagic strokes, with atherosclerosis and vascular wall mineralization identified as the primary underlying causes [6]. There is also a reported case in a white‐handed gibbon ( *Hylobates lar* ) [7]. In nonhuman primates, as in humans, the initial diagnosis is based on clinical signs, and imaging techniques, such as magnetic resonance imaging, can aid in confirmation. Although treatment is complex, it is possible; one report describes a chimpanzee that showed clinical improvement and stability for 6 years following treatment with anticoagulants and corticosteroids [8].

Arteriosclerosis observed in the blood vessels of the brain and other organs may have predisposed the animal from our report to thrombosis and subsequent stroke. The mild neutrophilic infiltrate in the brain was interpreted as a secondary reaction to the necrosis, given that the animal survived for at least 24 h after the onset of clinical signs. Furthermore, the absence of bacterial growth in culture and the negative Gram stain excludes the possibility of bacterial infections or septic thrombi. Neoplastic, cartilaginous, and bone emboli should also be considered in cases of infarction within the nervous system of animals, particularly in those with suspected intervertebral disc disease or trauma [9]. However, in the present case, no histological features suggestive of such origin were observed, and there were no additional lesions or clinical information to support this hypothesis.

Stroke should be considered in nonhuman primates with sudden neurological signs. Experimental studies aid in identifying clinical signs, imaging, and pathological findings for accurate diagnosis [2, 3, 4, 5]. Additionally, human literature is useful, as features like sudden onset, limb weakness or paralysis, and abnormal eye movements are common in acute ischemic stroke in humans [10].

## Funding

Work in ARO lab is supported by CNPq (Conselho Nacional de Desenvolvimento Científico e Tecnológico, Brazil), FAPEMIG (Fundação de Amparo à Pesquisa do Estado de Minas Gerais, Brazil), and CAPES (Coordenação de Aperfeiçoamento de Pessoal de Nível Superior, Brazil).

## Conflicts of Interest

The authors declare no conflicts of interest.

## Data Availability

Data sharing not applicable to this article as no datasets were generated or analysed during the current study.
